# Histone Deacetylase Inhibitor Scriptaid Alleviated Neurological Dysfunction after Experimental Intracerebral Hemorrhage in Mice

**DOI:** 10.1155/2018/6583267

**Published:** 2018-08-12

**Authors:** Heng Yang, Wei Ni, Hanqiang Jiang, Yu Lei, Jiabin Su, Yuxiang Gu, Liangfu Zhou

**Affiliations:** Department of Neurosurgery, Huashan Hospital, Fudan University, Shanghai, China

## Abstract

**Objectives:**

To investigate the role of Scriptaid in reducing brain injury after intracerebral hemorrhage (ICH) in mice.

**Methods:**

An ICH model was constructed by injecting autologous blood into the right basal ganglia in mice. The animals were administered 3.5 mg/kg of Scriptaid intraperitoneally after ICH. The hematoma volume and hemoglobin level were measured to examine hematoma resolution. A behavior test and brain edema and white matter injury examinations indicated brain injury after ICH.

**Results:**

Scriptaid treatment promoted hematoma resolution and reduced the hematoma volume 7 d after ICH compared with the vehicle group (*P* < 0.05). Scriptaid treatment also alleviated the brain water content in the ipsilateral basal ganglia (*P* < 0.05) and cortex (*P* < 0.01) 3 d after ICH. In addition, Scriptaid improved neurological function recovery and alleviated white matter injury 35 d after ICH.

**Conclusions:**

Scriptaid can protect against brain injury after ICH and may be considered a new medical therapy method for ICH.

## 1. Introduction

Intracerebral hemorrhage (ICH) is a fatal subtype of stroke with high morbidity and mortality that accounts for 10%–15% of all strokes [1]. The mechanisms of brain injury following ICH have achieved great understanding in past decades, including primary and secondary brain injuries. Primary brain damage is mainly due to the mass effect and mechanical disruption from extravasated blood. Secondary brain injury is much more complicated and is mainly caused by the physiological response to the hematoma and toxic blood components [2–4]. Unfortunately, no effective medical treatment is available to improve functional outcomes in patients with ICH.

Neuroinflammation plays a key role in secondary brain injury after ICH and is associated with both brain damage and recovery from ICH [5]. First, as resident immune cells in the central nervous system (CNS), activated microglia release many inflammatory factors. Then, systemic immune cells infiltrate and produce cytokines, chemokines, extracellular proteases, and reactive oxygen species (ROS) and induce a severe inflammatory reaction [6–9] after ICH. When microglia/macrophages are activated, they switch into two phenotypes (the M1 and M2 phenotypes) through the so-called microglia/macrophage polarization process [10]. Many previous studies have demonstrated that microglia/microphage polarization is dynamic during a central nervous system injury and modulating microglia/macrophage polarization ameliorates inflammatory injuries after traumatic brain injury (TBI) and ischemic and hemorrhagic strokes [11–13]. Recent studies have also demonstrated that activating microglia/macrophages enhances hematoma resolution and improves functional outcomes in a rat model of ICH [14, 15]. In addition, blood-brain barrier (BBB) disruption, which contributes to the development of perihematomal edema, is an important cause of secondary injury after ICH [3, 16].

Histone deacetylases (HDACs) are a class of enzymes that remove acetyl groups (O = C − CH_3_) from an *ε*-N-acetyl lysine amino acid on a histone, thereby allowing the histones to wrap the DNA more tightly. Histone deacetylase inhibitors (HDACis) have been widely used for the treatment of epilepsy, neurodegenerative diseases, and cancer [17–19]. Recently, significant progress has been made in increasing the understanding of the roles of HDACs in regulating inflammation and immunoreactions. Therapeutic application of HDACis for inflammatory and infectious diseases has also been described in many previous reports [20, 21]. In addition, an effort has been made to develop HDACis for the treatment of traumatic brain injury and stroke. For example, HDACis suppress immune activation and alleviate brain injury in TBI and ischemic stroke in animal models [22–25]. Scriptaid is a novel histone deacetylase inhibitor, and recent studies have indicated that Scriptaid modulates microglia/macrophage polarization and protects against traumatic brain injury [25, 26]. However, few studies have investigated the application of Scriptaid in therapy for intracerebral hemorrhage. Therefore, the present study investigated the effect of Scriptaid on the amelioration of brain injury in a mouse ICH model.

## 2. Materials and Methods

### 2.1. Animals and ICH Model Construction

C57BL/6 male mice (8–10 weeks, 20–26 g) were purchased from the Chinese Academy of Sciences at Shanghai. All animals were provided free access to food and water, and all experiments were approved by the Animal Ethics Committee of Fudan University. Moreover, all experiments were performed and reported according to the ARRIVE guidelines (http://www.nc3rs.org.uk/arrive). ICH model construction was established previously [27, 28]. Briefly, animals were intraperitoneally anesthetized with 4% chloral hydrate. Blood was obtained from a femoral artery catheter for analysis of pH, PaO_2_, PaCO_2_, and blood glucose. The core body temperature was maintained at 36.0 ± 1.0°C with a feedback-controlled heating pad. The physiological information is shown in Table 1. The mice were positioned in a mouse stereotaxic frame, and 30 *μ*l of nonanticoagulated autologous blood obtained from the femoral artery of the mouse or saline was injected straight into the right basal ganglia (0.2 mm anterior, 3.5 mm ventral, and 2.5 mm lateral to the bregma) at a rate of 2 *μ*l/min with a microinfusion pump. The needle remained in position for an additional 10 minutes after the blood was fully injected and then was removed gently. The burr hole was filled with bone wax, and the skin incision was sutured.

### 2.2. Experimental Groups

The animals were divided into three groups: sham, ICH + vehicle, and ICH + Scriptaid (3.5 mg/kg). Animal group assignments for the ICH or sham operation, treatment with Scriptaid or vehicle, and neurobehavioral testing or sacrifice for hematoma measurement, brain water content assessment, and immunostaining were performed randomly using a lottery drawing box. All main outcome studies, including neurobehavioral tests, hematoma volume, brain water content, and immunohistochemistry, were performed by investigators blinded to the group assignments and experimental conditions. As described previously [25], the animals were administered 3.5 mg/kg of Scriptaid intraperitoneally at 2, 26, and 50 hours after ICH. The ICH + vehicle group was treated with an equivalent volume and concentration of dimethyl sulfoxide diluted in saline on the same time course. Then, behavioral testing was performed on the animals, and the animals were euthanized at days 1, 3, 7, and 35 after ICH for hematoma measurement, brain water content calculation, and immunohistochemistry assays.

### 2.3. Hematoma Measurement

As described previously [29], the brains were removed after perfusion and sliced into 1 mm-thick sections. ImageJ (version 1.49, NIH) was used to measure the ICH volumes. Additionally, the hemoglobin levels in the brain tissues were measured to quantify the hematoma size [15]. After ICH, the contralateral hemisphere and ipsilateral cortex were removed and the remaining brain tissue was dissolved in Drabkin's reagent. Then, the supernatant of the homogenate was collected and measured using a spectrophotometer. The hematoma volume (microliters) was calculated using the standard curve.

### 2.4. Behavior Tests

The behavior tests included the corner turn test and the wire hanging test, which were described previously [25, 26, 30]. Before the behavior tests, all animals underwent behavior training for three days and abnormal animals were excluded. The corner turn test was performed as follows. Mice were allowed to proceed into a corner with a 30° angle. The directions (left or right) of the turns made by the mice were recorded. Every mouse repeated this procedure 20 times. The percentage of right turns was calculated. The wire hanging apparatus was a stainless steel bar (50 cm length, 2 mm diameter) resting on two vertical supports and elevated 37 cm above a flat surface. Mice were placed on the middle of the bar and were observed for 30 seconds in 4 trials. The amount of time spent hanging was recorded and scored according to the following system: 0, fell off; 1, hung onto the bar with two forepaws; 2, hung onto the bar with added attempt to climb onto the bar; 3, hung onto the bar with two forepaws and one or two hind paws; 4, hung onto the bar with all four paws and with tail wrapped around the bar; and 5, escaped to one of the supports. All behavior tests were performed and evaluated by a blinded observer.

### 2.5. Brain Water Content

The brain water content measurement was performed as previously reported [31]. After euthanization and decapitation, the mouse brains were removed and divided into the following five parts: the ipsilateral cortex, ipsilateral basal ganglia, contralateral cortex, contralateral basal ganglia, and cerebellum. Every brain part was weighed with an electronic balance to obtain the wet weight and then dried in a gravity oven at 100°C for 24 hours to obtain the dry weight. The brain water content was calculated with the following formula: brain water content (%) = (wet weight–dry weight)/wet weight∗100%.

### 2.6. Immunohistochemistry Staining

Brains were removed after perfusions with saline and 4% paraformaldehyde (Sigma-Aldrich, St. Louis, MO, USA) and gradient dehydrated with 20% and 30% sucrose in PBS. Then, the sample brains were sectioned into 25 *μ*m-thick sections on a freezing microtome and subjected to immunohistochemistry. Immunohistochemistry staining was performed as previously described [12]. The primary antibodies included rabbit anti-MBP (myelin basic protein) (Abcam, 1 : 500 dilution, Cambridge, MA, USA) and mouse antinonphosphorylated neurofilaments (SMI-32, 1 : 500 dilution, Abcam). Alexa Flour conjugated antibodies (Invitrogen, Grand Island, NY, USA, 1 : 1000) were used as the secondary antibodies. The slides were covered with Fluoroshield with DAPI (Sigma-Aldrich, St. Louis, MO, USA). Double labeling was analyzed using an epifluorescence microscope.

### 2.7. Statistical Analysis

All data in this study are presented as the mean ± standard error of the mean (SEM) and have been analyzed using the SPSS 19.0 software. Student's *t*-test was used to analyze differences between two groups, whereas differences between multiple groups were analyzed with one-way ANOVA. Two-way ANOVA was used to evaluate differences in the behavior tests between groups and between time points. Differences were considered significant when *P* < 0.05.

## 3. Results

### 3.1. Scriptaid Improved Hematoma Resolution after Intracerebral Hemorrhage

**Figure 1:**
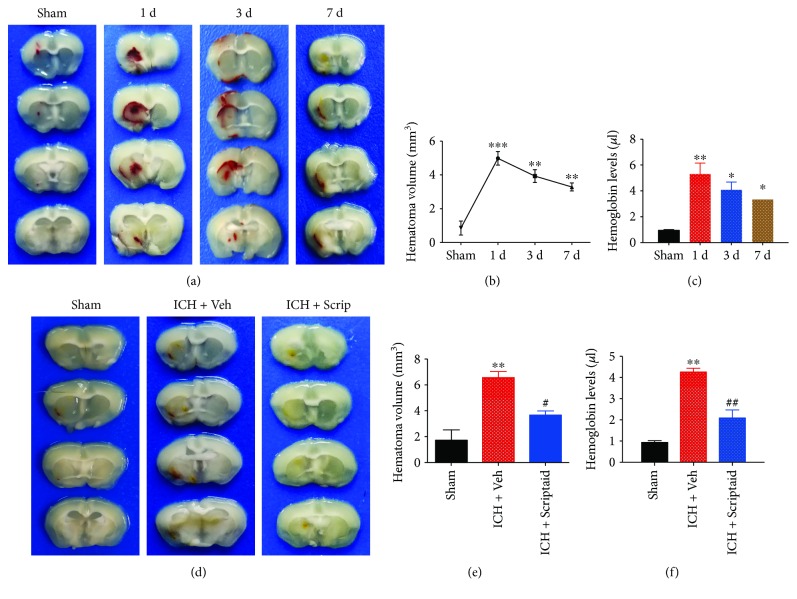
Scriptaid promoted hematoma resolution in an experimental intracerebral hemorrhage model. (a) Representative images of hematoma volumes at 1 d, 3 d, and 7 d after ICH. Time course of hematoma volume (b) and hemoglobin level (c) changes after ICH (^∗^*P* < 0.05, ^∗∗^*P* < 0.01, and ^∗∗∗^*P* < 0.001). (d) Serial coronal sections of mouse brain tissues at 7 d after ICH. The hematoma volumes (e) and hemoglobin levels (f) at 7 d after ICH (^∗∗^*P* < 0.01 versus the sham group; ^#^*P* < 0.05 versus the vehicle group).

The hematoma mass effect is an important factor for brain injury after ICH. The hematoma morphology and size at 1, 3, and 7 d post-ICH are demonstrated in Figure 1(a) and show the hematoma absorption process (Figure 1(b)). In addition, the hemoglobin level indirectly demonstrated dynamic changes in the hematoma volume (Figure 1(c)). Compared with the vehicle group, Scriptaid treatment improved hematoma absorption at 7 d post-ICH (Figure 1(d)). Scriptaid treatment reduced the hematoma volume by 44% (3.29 ± 0.41 mm^3^ in the vehicle group versus 1.85 ± 0.32 mm^3^ in the Scriptaid group; *P* < 0.05) at 7 d post-ICH (Figure 1(e)). The hemoglobin level also decreased by 44.4% (3.73 ± 0.77 *μ*l in the vehicle group versus 2.08 ± 0.68) at 7 d post-ICH, although the difference was not significant (Figure 1(f)).

### 3.2. Scriptaid Improved Neurological Dysfunction after Intracerebral Hemorrhage

**Figure 2:**
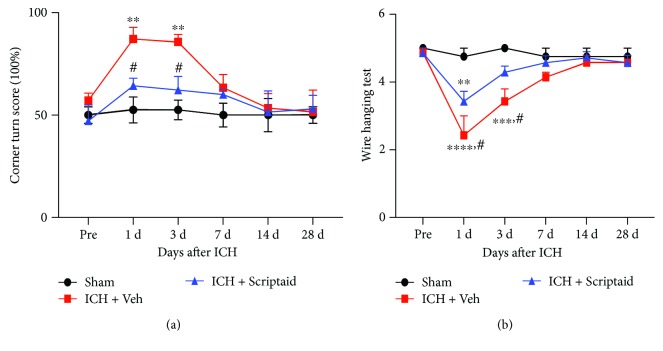
Scriptaid improved the neurological function deficit after ICH. (a) Corner turn test: Scriptaid treatment alleviated sensory deficits at 1 d and 3 d after ICH compared with the ICH + vehicle group (^∗∗^*P* < 0.01 versus the sham group; ^#^*P* < 0.05 versus the ICH + vehicle group). (b) Wire hanging test: Scriptaid treatment alleviated motor deficits at 1 d and 3 d after ICH compared with the ICH + vehicle group (^∗∗^*P* < 0.01, ^∗∗∗^*P* < 0.001, and ^∗∗∗∗^*P* < 0.001 versus the sham group; ^#^*P* < 0.05 versus the ICH + vehicle group).

Scriptaid treatment ameliorated both sensorimotor functions after ICH. In the corner turn test, Scriptaid administration reduced the corner turn score significantly at 1 and 3 d post-ICH (Figure 2(a), *P* < 0.05). Additionally, the wire hanging test showed motor deficit improvement in the Scriptaid treatment group at 1 and 3 d post-ICH compared with the vehicle group (Figure 2(b), *P* < 0.05).

### 3.3. Scriptaid Reduced the Brain Water Content 3 Days after Intracerebral Hemorrhage

**Figure 3:**
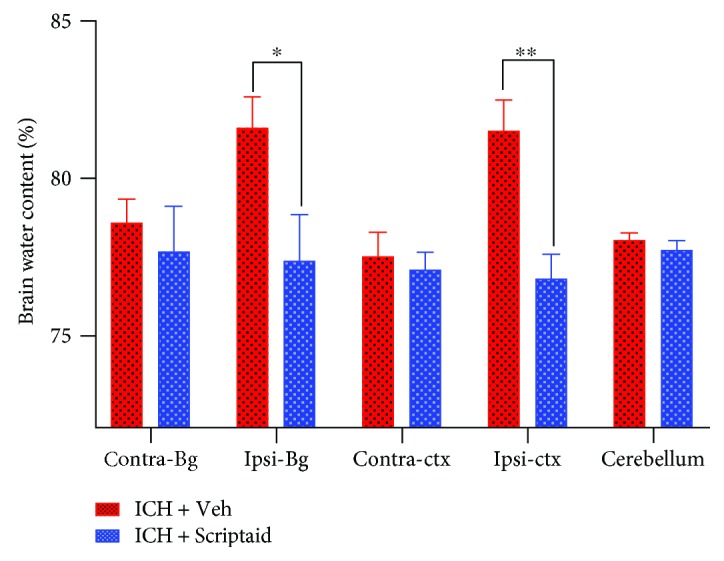
Scriptaid reduced the brain water content at 3 d after ICH. Compared with the vehicle group, the Scriptaid treatment group exhibited a significantly reduced brain water content in the ipsilateral basal ganglia and ipsilateral cortex at 3 d after ICH (^∗^*P* < 0.05, ^∗∗^*P* < 0.01 versus the vehicle group). No significant difference in the brain water content in the contralateral basal ganglia and cortex was found between the Scriptaid treatment group and the vehicle group.

Scriptaid treatment reduced brain edema in the ipsilateral basal ganglia (brain water content 81.53 ± 1.68 versus 77.31 ± 2.52 in the vehicle-treated controls, *P* < 0.05) and ipsilateral cortex (brain water content 81.43 ± 1.72 versus 76.74 ± 1.34 in the vehicle-treated controls, *P* < 0.01) at 3 d post-ICH. However, no significant difference was found in the brain water content in the contralateral basal ganglia and cortex between the Scriptaid treatment group and the vehicle group (Figure 3).

### 3.4. Scriptaid Conferred Long-Term Preservation of White Matter after ICH

**Figure 4:**
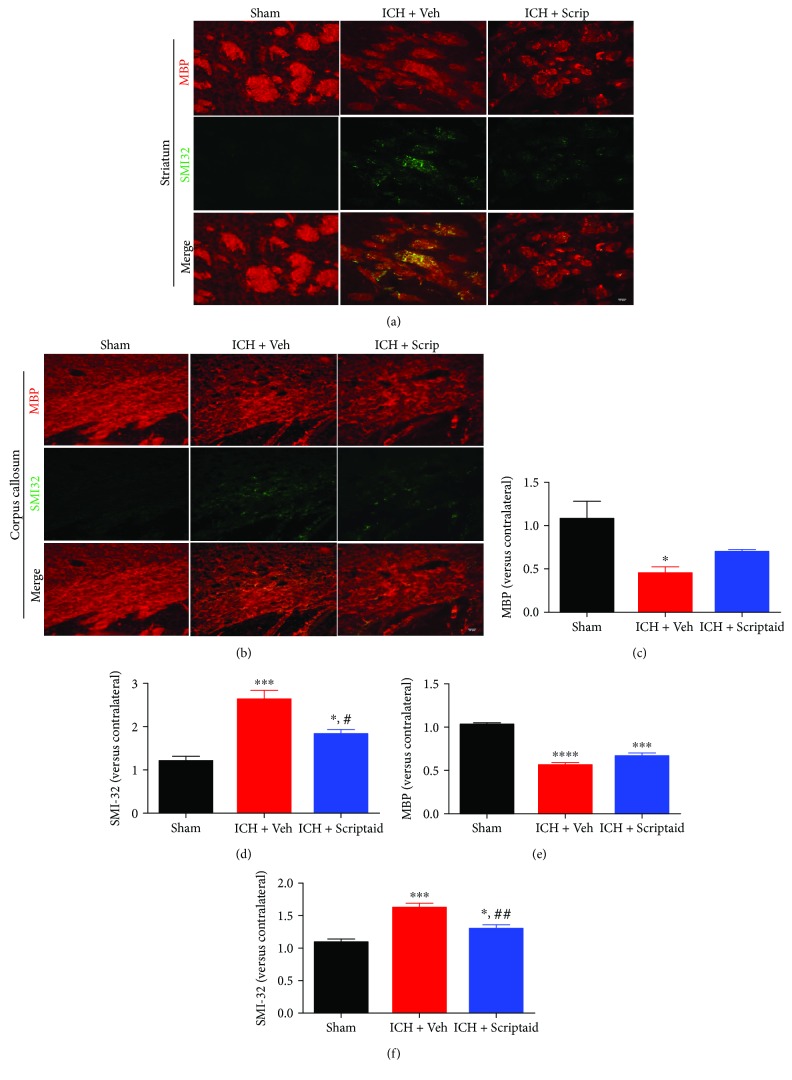
Scriptaid alleviated white matter injury at 35 d after ICH. (a, b) Representative images of myelin basic protein (MBP) (red) and SMI32 (green) immunostaining in the striatum (a) and corpus callosum (b) (scale bar = 25 *μ*m). (c, d) The relative ratio of ipsilateral versus contralateral MBP (c) and SMI32 (d) immunostaining intensity 35 d after ICH in the striatum. (e, f) The relative ratio of ipsilateral versus contralateral MBP (e) and SMI32 (f) immunostaining intensity 35 d after ICH in the corpus callosum (^∗^*P* < 0.05, ^∗∗∗^*P* < 0.001, and ^∗∗∗∗^*P* < 0.0001 versus the sham group; ^#^*P* < 0.05 and ^##^*P* < 0.01 versus the vehicle group).

White matter injury was evaluated by double immunofluorescent staining for MBP and SMI-32 in the ipsilateral striatum and corpus callosum at 35 d post-ICH (vehicle and Scriptaid groups) or after sham surgery (sham group) (Figures 4(a) and 4(b)). In the sham group, SMI-32 immunoreactivity and high MBP expression were rarely observed in the ipsilateral striatum and corpus callosum, which demonstrated no white matter injury. Scriptaid failed to alleviate MBP expression at 35 d post-ICH in the ipsilateral striatum and corpus callosum compared with those in the vehicle group (Figures 4(c) and 4(e)). However, Scriptaid treatment reduced SMI-32 expression at 35 d post-ICH, which demonstrated alleviation of white matter injury. The relative ratio of the ipsilateral versus contralateral SMI-32 immunostaining intensity was lower in the Scriptaid group than in the vehicle group (1.83 ± 0.14 versus 2.64 ± 0.34 in the striatum, *P* < 0.05; 1.30 ± 0.09 versus 1.62 ± 0.10 in the corpus callosum, *P* < 0.01; Figures 4(d) and 4(f)).

## 4. Discussion

Although many previous studies have described the mechanism of ICH and have attempted to find a therapy, no effective medical treatment for ICH is available at present. HDACis have been shown to possess neuroprotective effects in animal models of TBI and stroke [22–25]. As a novel histone deacetylase inhibitor, Scriptaid was demonstrated to protect against white matter injury in a mouse TBI model. However, the neuroprotective effects of Scriptaid in ICH remain unknown. In the present study, we found that Scriptaid improved hematoma resolution and alleviated brain edema after ICH. In addition, Scriptaid treatment had an effect on preserving white matter integrity at 35 d post-ICH and promoting neurological behavioral functions, thereby facilitating long-term functional recovery after ICH.

Secondary brain injury due to ICH is mainly caused by direct toxicity and inflammation induced by toxic blood components and hematoma metabolic products. Therefore, hematoma removal is an important target for ICH treatment because this process can relieve mass effects, alleviate inflammation, and improve the recovery of neuronal function [14, 15, 29]. In our study, Scriptaid improved hematoma resolution through a mechanism that might be related to modulation of microglia/macrophage polarization. As a novel histone deacetylase inhibitor, Scriptaid can shift microglia/macrophage polarization toward the M2 phenotype and mitigate inflammation [25]. Microglia/macrophages play a significant role in hematoma clearance. Recent studies indicated that activating microglia/macrophages promoted hematoma absorption and neurological outcomes after ICH [14, 15, 28, 32]. However, the specific mechanism by which Scriptaid treatment improves hematoma resolution remains unknown and requires further research. In mouse TBI models, 3.5 mg/kg is the optimal dose of Scriptaid for TBI treatment [26]. Although many differences in the mechanism of brain injury exist between TBI and ICH, both conditions involve hematoma and brain edema. Therefore, we also chose 3.5 mg/kg for the treatment of ICH in our study.

Perihematomal brain edema can occur within hours of ICH and peak at several days [33, 34]. Brain edema after ICH can increase intracranial pressure and induce herniation. Edema formation after ICH has several phases, including an early phase (involving clot retraction and serum protein accumulation around the hematoma), a second phase (involving thrombin), and a third phase (involving erythrocyte components) [3, 33]. In the present study, Scriptaid treatment reduced perihematomal brain tissue edema at 3 d post-ICH. Thus, Scriptaid can improve hematoma clearance and reduce the mass effect, whereas HDACis can reduce inflammation and then protect the blood-brain barrier from disruption in mouse and rat ICH models [23, 24]. White matter injury is another important reason for poor outcomes of ICH. Recently, significant progress has been made in increasing the understanding of the role of white matter injury after ICH [35, 36]. Consistent with the present finding, Scriptaid has been described to prevent white matter injury in mouse TBI models [25]. Our data also showed that Scriptaid reduced white matter injury in mouse ICH models. Moreover, reducing white matter after ICH could improve neurological behavioral functions, such as sensorimotor function.

Although this study is a novel evaluation focused on the preservation of brain injury in ICH by Scriptaid, there are still several limitations: (1) no information was provided about the anti-inflammatory effect of Scriptaid and (2) no data demonstrated the relationship between microglia/macrophage polarization and hematoma resolution after ICH. Therefore, we will conduct further research on the mechanism by which Scriptaid preserves brain injury in ICH.

In conclusion, our data demonstrate that Scriptaid treatment protects against brain injury by improving hematoma resolution, reducing brain edema, and alleviating white matter injury in a mouse ICH model. Thus, the histone deacetylase inhibitor Scriptaid can be considered a new medical therapy method for ICH.

## Figures and Tables

**Table 1:** General physiological information for the different groups.

Group	MABP (mmHg)	pH	PO_2_ (mmHg)	PCO_2_ (mmHg)	Hct. (%)	Glucose (mg/dl)	Core body temperature
Sham	72.1 ± 7.9	7.42 ± 0.03	98.3 ± 4.1	42.3 ± 4.3	41.3 ± 0.5	91.5 ± 11.2	36.2 ± 0.4
ICH + vehicle	73.5 ± 8.4	7.36 ± 0.02	96.9 ± 2.4	41.5 ± 3.1	41.8 ± 1.1	92.3 ± 8.1	36.5 ± 0.2
ICH + Scriptaid	73.2 ± 9.1	7.38 ± 0.04	97.3 ± 2.8	42.1 ± 2.5	42.1 ± 0.8	94.2 ± 10.5	36.1 ± 0.8

## Data Availability

The data used to support the findings of this study have been deposited in the figshare repository (https://figshare.com/s/ff7cca2d39bb5fa3839b).
